# CerviCell-detector: An object detection approach for identifying the cancerous cells in pap smear images of cervical cancer

**DOI:** 10.1016/j.heliyon.2023.e22324

**Published:** 2023-11-14

**Authors:** Madhura Kalbhor, Swati Shinde, Pankaj Wajire, Hemanth Jude

**Affiliations:** a Pimpri Chinchwad College of Engineering, Pune, India; b Karunya Institute of Technology and Sciences, India

**Keywords:** Cervical cancer, Pap smear, Artificial intelligence, Object detection, YOLOv5, Faster R–CNN, Detectron2

## Abstract

Cervical cancer is the second most commonly seen cancer in women. It affects the cervix portion of the vagina. The most preferred diagnostic test required for screening for cervical cancer is the pap smear test. Pap smear is a time-consuming test as it requires detailed analysis by expert cytologists. Cytologists can screen around 100 to 1000 slides depending upon the availability of advanced equipment. It requires substantial time and effort to carefully examine each slide, identify and classify cells, and make accurate diagnoses. Prolonged periods of visual inspection can increase the likelihood of human errors, such as overlooking abnormalities or misclassifying cells. The sheer volume of slides to be screened can exacerbate fatigue and impact diagnostic accuracy. Due to this reason Artificial intelligence (AI) based computer-aided diagnosis system for the classification and detection of pap smear images is needed. There are some AI-based solutions proposed in the literature, still, an effective and accurate system is under research. In this paper, we implement a state-of-the-art object detection model with a newly available CRIC dataset which follows the Bethesda system for nomenclature. Object detection models implemented are YOLOv5 which uses the CSPNet backbone, Faster R–CNN which has Region Proposal Network (RPN) and Detectron2 framework created by Facebook AI Research (FAIR) Group. ResNext model is implemented among the available models from Detectron2. The CRIC dataset is preprocessed and augmented using Roboflow tool. The performance measures of Average Precision and mean Average precision over the Intersection over Union (IoU) are used to evaluate the effectiveness of the models. The models performed better for two classes namely Normal and Abnormal compared to six classes from the Bethesda system. The highest mean Average Precision (mAP) is observed on the augmented dataset for YOLOv5 models for binary classification with 83 % mAP with IoU in the range of 0.50–0.95.

## 1 Introduction

Cervical cancer is one of the leading causes of death among women. This cancer infects cervix tissue in the lowermost part of the uterus. Cervix connects the main body of the uterus and the vagina acting as a gateway. A cancerous virus at the cervix damages the tissue, which could lead to medical complications. Cervical cancer can be fatal if not treated at an early stage. Still, in low and middle-income countries, due to lack of infrastructure, testing for cervical cancer is not very common, and potential patients go unnoticed [1]. A Pap smear test is a primary screening method for finding precancerous or cancerous cells [2]. A Pap smear test requires a medical expert to take samples from the cervix surface, and this sample is then sent to the pathology lab. The sample is viewed under a microscope. Pathologists are required to detect and classify each cell from the slide of a microscope which contains hundreds of cells. Detection of cancerous or precancerous cells remains subjective, depending on the expertise of pathologists.

Advancements in computer vision technology have given an edge over conventional practices in medical imaging [3]. Using computer vision, tasks are automated, and artificial intelligence is used for prediction. From images, the models learn features and use this for the accurate detection of cells. The larger the image dataset, the more a variety of features are learned by the model, which ultimately transforms into better prediction. In this paper, we have developed state-of-the-art object detection models, namely YOLOV5 [4], Faster RCNN [5], and Detectron2 [6], using pap-smear images along with augmentation of input training images for the increasing training set. YOLOV5 is the latest version of the YOLO series, which is an extension of YOLOV3 using the PyTorch framework, while the YOLOV4 version is built using the darknet framework. Faster RCNN is an extension of Fast RCNN; it uses a region proposal network (RPN) and anchor boxes to give faster output. The computation required for convolutional is shared across the RPN and Fast RCNN module present in the Faster RCNN architecture. Detectron2 is another computer vision model written in PyTorch by Facebook AI Research Group (FAIR). In this paper, these models are being used for the prediction of the pap smear dataset that contains a variety of images collected from frames of slides under the microscope.

The objectives of the paper are as follows.

1. The paper addresses the task of classifying cell images into six distinct classes, including Atypical Squamous Cells of Undetermined Significance(ASC-US), SCC, Lesions of low-grade squamous intraepithelial lesion (LSIL), Atypical Squamous Cells high-grade squamous intraepithelial lesion cannot be excluded (ASC-H), high-grade squamous intraepithelial lesion HSIL (abnormal classes), and Negative for Intraepithelial Lesion or Malignancy NILM (normal class). This classification task is crucial in various medical applications, particularly in the field of cytology and pathology.
2. The paper explores the use of three popular object detection models, namely YOLOv5, Faster RCNN, and Detectron2, for the classification task. These models are well-known in the computer vision community and have been extensively used for various object detection tasks. The choice of different models allows for a comprehensive comparison of their performance in classifying the cell images.
3. The paper investigates the impact of data augmentation on the classification performance of object detection models. Data augmentation involves applying various transformations to the input images, thereby increasing the diversity of the training data. The paper aims to improve the models' generalization and robustness.
4. The paper conducts a comparative analysis of the three object detection models, both with and without augmentation. By evaluating their performance on the cell image classification task, the paper provides insights into the strengths and weaknesses of each model. This analysis can aid researchers and practitioners in selecting the most suitable model for similar classification tasks in the medical domain.

## 2 Literature survey

An automated screening method for pap smear cervical cancer using deep learning techniques has been experimented by many researchers across the world. Different object detection models have been tried on various medical images like X-rays, radiography, MRIs, CT scans, and many more [7]. The use of deep learning for medical imaging purposes has proved to be a boon, helping faster testing and diagnosis, thus saving lives. Similarly, for cervical cancer screening, deep learning techniques like classification and detection have shown good results. Table 1 refers to the authors and the approach they used for object detection.

**Table 1:** Literature survey.

Authors	Dataset	Method	Description
Xia Li et al. [7]	Digital Human Body” (DHB) Vision Challenge-Intelligent Diagnosis of Cervical Cancer Risk provided by the Alibaba Cloud TianChi Company	Deformable and Global Context-Aware Faster RCNN-FPN	To boost scalability, the authors implemented a faster RCNN-FPN model created by including deformable convolution layers into the feature pyramid network (FPN). But the Images were of the Whole Slide, which is too specific for the generic use case.
Lei Cao et al. [8]	Heilongjiang Maternal and Child Health Hospital (HMCHH dataset) and Harbin Medical University Cancer Hospital (HMUCH dataset)	Attention-guided convolutional network	Authors proposed a novel method named attention feature pyramid network (AttFPN) based on the feature pyramid network (FPN) and attention mechanism [9]. But the proposed model only dealt with abnormal cervical cells.
M. B. Bijoy et al. [10]	International Skin Imaging Collaboration (ISIC) lesions dataset and MobileODT cervical data.	Self-supervision boosted object detection technique.	The authors implemented FasterRCNN and an ensembledEfficientNet B4 for cervix type detection and classification. They achieved a 0.623 score for Intersection over Union (IoU).
Mingyang Xia et al. [11]	Peking Union Medical College Hospital and Ningbo Konfoong Biotech International Co., Ltd	Series-parallel fusion network (SPFNet).	The proposed framework obtained 78.4 % AP in cervical cancer cell detection tasks. It uses a binary class dataset with Normal and Abnormal classes.
Karasu Benyes et al. [12]	SurePath datasets LBC Dataset	ResNet50, DenseNet121, ResNet152, ResNet101, EfficientNetB0 an ensemble, a novel convolutional neural network (CNN), and a CNN + autoencoder (AE).	Five transfer learning models, an ensemble, a novel CNN, and a CNN + AE achieved >90 % accuracy classifying SurePath images. The AE CNN model, 99.80 % smaller than average transfer models, maintained 96.54 % accuracy. ThinPrep Pap classification accuracies were lower but improved with Deep CORAL domain adaptation, with ResNet101 achieving the highest accuracy at 92.65 %
Alsalatie, Met al. [13]	LBC Dataset.	Ensemble deep learning model.	The automatic diagnosis of WSI is an ensemble deep learning model. Up to 99.6 % accuracy is achieved in the suggested network's classification between four classes. It focuses on the full stained slice image rather than just one particular cell.
Hussain, Elima et al. [14]	LBC Dataset.	Fusion-based decision from ensemble deep convolutional neural network.	The proposed method is evaluated using three datasets: liquid-based cytology, conventional and Herlev datasets where the better result is reported by the ensemble classifier with 0.989 accuracy, 0.978 sensitivity and 0.979 specificity.
Subarna, Tet al. [15]	LBC Dataset	CEENET Model	The proposed approach for cervical image classification consists of multiple modules: Edge detector (Kirsch's edge detector) to identify edges in the source cervical image, Complex Wavelet Transform (CWT) to decompose the edge-detected images into sub-bands, and the computation of Local Derivative Pattern (LDP) and statistical features from these sub-bands. A feature map is constructed using these computed features along with the source cervical image. The Cervical Ensemble Network (CEENET) model takes both the feature map and the source cervical image as input for the final classification task, distinguishing between healthy and cancer-affected cervical images.

## 3 Methodology

**Fig. 1:**
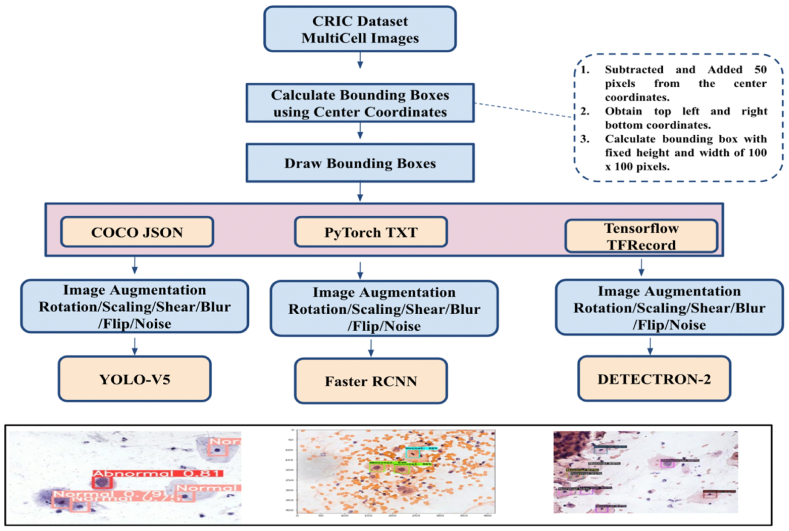
Proposed Methodology.

Fig. 1 shows the overall methodology used to carry out the experimentations. The data is collected form CRIC dataset for which the bounding boxes using the centre coordinates are calculated. The bounding boxes are represented in different formats compatible with the different detection algorithms. Data augmentation is performed and different detection objects like YOLO-V5, Faster CNN and Detectron-2 are experimented to detect the normal and abnormal cells. In detail, each step is discussed in the following sections.

### 3.1 Data collection

An important part of an automated system powered by computer vision and deep learning models is a large dataset of good quality, well-curated digitised images, and ground truth. The dataset available that are findable, accessible, interoperable, and reusable are limited. Some of the public datasets on pap smear are Herlev [16,17] and Sipakmed [18,19], but they have a low quality of images and do not follow the Bethesda system of nomenclature [20], making it redundant for the real-time use case. The dataset required for training and testing the object detection model that we built is collected from the Center for Recognition and Inspection of Cells (CRIC). CRIC is a searchable cell image database [21]. The images on CRIC searchable image database are collected by biology and computer science researchers. The photo documentation for CRIC Database was done following conventional microscopy in the light field with an objective lens of 40× times; and an ocular lens of 10× times. The digital camera used was Zeiss AxionCam MRC attached to a microscopy Zeiss AxioImager.Z2 powered by Software Axion Vision Zeiss. CRIC dataset follows the Bethesda system, which is considered the most uniform and reproducible terminology among various pathologists.

### 3.2 Pre-processing of dataset

**Fig. 2:**
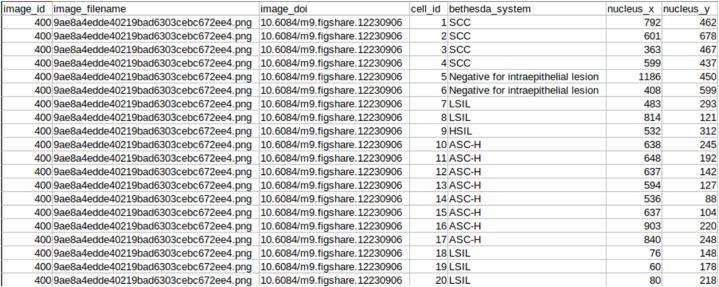
Snapshot of CSV file of the Dataset.

**Fig. 3:**
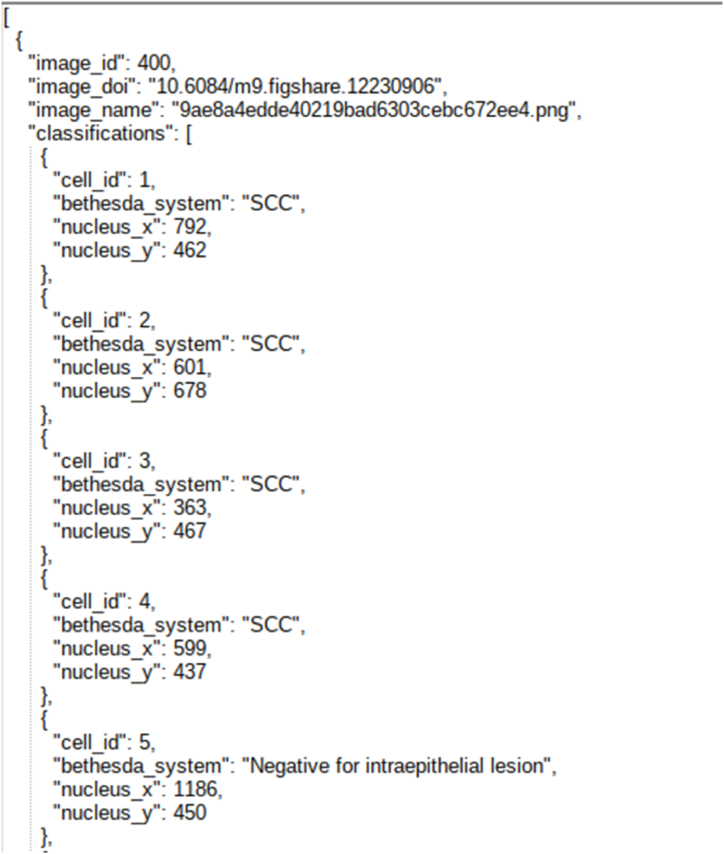
Snapshot of JSON file of the Dataset.

For an object detection task, images and ground truth are equally important. The images and ground truth available in CRIC searchable image database is downloaded where images are in Portable Network Graphics (PNG) format, and ground truth is available in CSV and JSON file formats. The ground truth file contains classifications of the cells. Fig. 2 shows the CSV file with column names. Fig. 3 shows the cell classification details in JSON file format. From the classification information, we gained the centre coordinates of the nucleus of each cell. We calculated a bounding box with a constant height and width of 100 pixels × 100 pixels using these centre coordinates.

### 3.3 Bounding boxes and ground truth formats

**Fig. 4:**
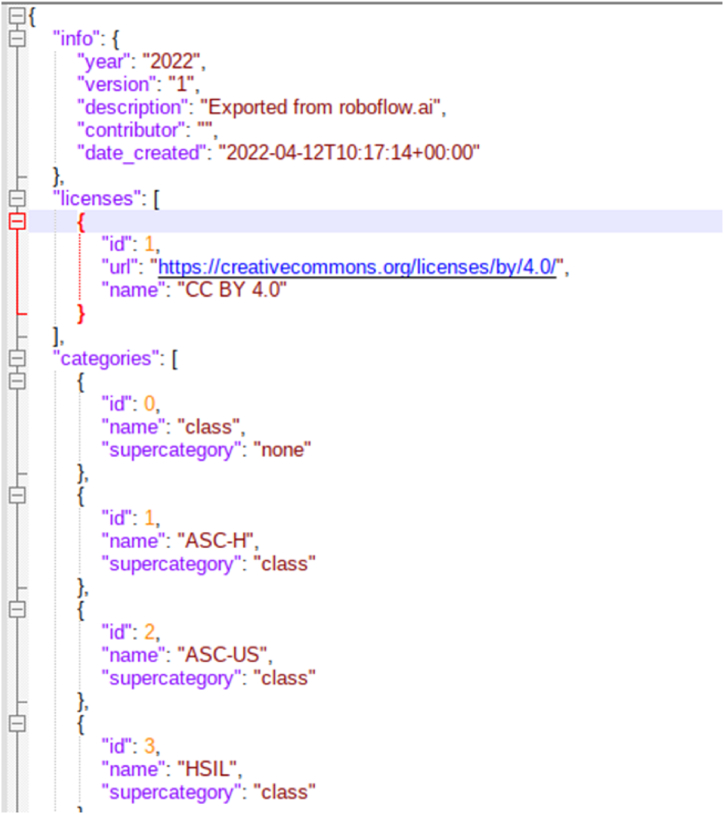
COCO annotation data sample for our dataset.

**Fig. 5:**
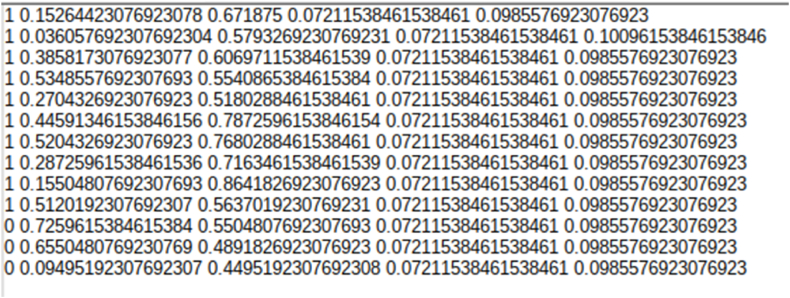
Sample annotation of the PyTorch TXT file.

In object detection, we use bounding boxes to describe the location of an object in the spatial field. It is a rectangular-shaped box that acts as a reference for object detection. The metadata provided by CRIC with the classification provided a way to represent this bounding box utilising the centre coordinates. These bounding boxes are drawn using data annotators. These bounding boxes can be expressed in multiple formats. Formats utilised by models that we experimented with are COCO JSON, Pytorch TXT format generated from Pascal VOC, and TensorflowTFRecord. Microsoft's Common Objects in Context dataset (COCO) is one of the popular object detection datasets [22]. The format used in the dataset for storing annotation data is known as COCO, where data is stored in JSON format. COCO format contains annotations like bounding boxes, object class, and image metadata like height, width, and image source from the storage. Fig. 4 shows the COCO annotation data sample for our dataset. Detectron2 requires COCO JSON annotation files for creating bounding boxes. Another annotation format required by the Faster RCNN model for bounding boxes is TensorflowTFRecord. TensorflowTFRecord is a format that stores a sequence of binary records. It occupies less space than the original data, and also the reading operation can be done parallelly with I/O operations. It stores information in a single file meaning no two separate folders maintaining images and annotations are required. For reading bounding box annotation for the YOLOV5 model, we used the PyTorch TXT format [23]. This annotation format is similar to YOLO Darknet TXT. In addition to the TXT file, it contains the YAML file, which includes class values. The TXT file contains an object class denoted by an integer number and four float values depicting coordinates along with the width and height of the image. Fig. 5 shows a sample annotation of the PyTorch TXT file.

### 3.4 Ground truth conversion using roboflow

**Fig. 6:**
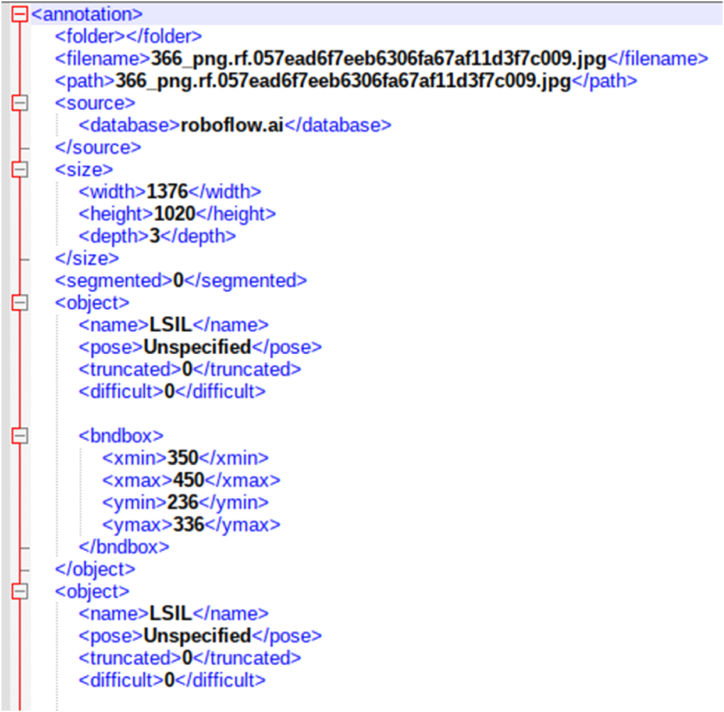
Pascal VOC format XML file of cell annotations from the CRIC dataset.

For utilising the CRIC dataset as mentioned earlier, we converted the centre coordinated into bounding boxes with constant width and height of 100 pixels × 100 pixels. For doing this, we subtracted and added 50 pixels value from the centre coordinate, by which we were able to obtain diagonal vertices coordinates of the bounding boxes. The CSV annotation file was used to perform these mathematical operations. After which, we converted the CSV annotation file to Pascal VOC format, which is more user-friendly and readable. Pascal VOC format uses XML for the representation of bounding boxes. This annotation format was originally created for the Visual Object Challenge (VOC) [24], after which it became a common interchange format for annotations of object detection. Fig. 6 shows the Pascal VOC format XML file of cell annotations from the CRIC dataset. We converted our annotation dataset into Pascal VOC XML format since the data format in Pascal VOC is the same as the annotation data we calculated after mathematical operations. Pascal VOC bounding boxes are represented by top-left and bottom-right coordinates. After converting annotation data into XML files, we used the Roboflow [25] tool to convert existing annotation formats to target annotation formats as per model needs. Roboflow provides easy to use annotation exporting method, reducing the tedious work of formatting datasets. The annotation conversion tool of Roboflow is free and user-friendly. The image files from the CRIC database and Pascal VOC XML files generated are uploaded on the tool. It reads the annotation for each image uploaded and marks it on the image. Once the upload is completed with annotations, the corresponding annotation format can be exported by the user by selecting the format from the menu. Using the Roboflow tool, we exported annotation data in PyTorch TXT files for the YOLOV5 object detection model, COCO JSON annotation files for the Detectron2 model, and TensorflowTFRecord file format for the Faster RCNN model.

### 3.5 Augmentation and pre-processing images

Apart from generating annotation, Roboflow can be used for pre-processing images like resizing, auto- orientation, and augmenting images to increase training data. Roboflow performs multiple operations on input image data like flipping, rotation, brightening, darkening, crop, shear, blur, and random noise, to name a few. We used Roboflow for pre-processing images so that the training, validation, and testing set are uniform and learning occurs on the same image properties. Depending on the model, we need to resize images to the same size that the model uses to learn and make an inference. We applied auto-orientation, which displays images in the same way they are stored on disk using Exchangeable Image File format. We resized images to 416 pixels × 416 pixels along with adjusting annotations accordingly. In the process of resizing, there is no loss in source image data. To increase size of dataset, we used Roboflow augmentation. Image augmentation helps to increase model performance by increasing the diversity of datasets for the models. In order to conduct our experiment, we augmented the CRIC dataset by flipping images both horizontally and vertically, then applying a hue and saturation operation with a range of −25 to +25° and deliberately adding salt and pepper noise to a limit of 5 % of pixels. A research study from Arizona State University [26] depicted the adverse effect noisy images have on accuracy classification. This makes the model more robust and similar to real-life pap smear samples. Table 2 below shows pre-processing and augmentation operations we performed on the CRIC dataset.

**Table 2:** Pre-processing and augmentation operations.

Task	Operations	Value/Range
Pre-processing	Auto-Orient	Applied
	Resize	Stretch to 416 × 416
Augmentations	Flip	Horizontal, Vertical
	Hue	Between −25° and +25°
	Saturation	Between −25 % and +25 %
	Noise	Up to 5 % of pixels

### 3.6 Models used

a. **Yolov5**

YOLO is an acronym for "You Only Look Once."It is an object detection algorithm that divides images into a grid system [27]. An object is then tried to be detected within the grid in YOLO. YOLOv1 was released in 2015 in a research paper titled "You Only Look Once: Unified, Real-Time Object Detection" [28]. Thereafter YOLOv2 [24] and YOLOv3 [29] were released in the year 2016 and 2018, respectively, by the original authors of YOLOv1. Later a new set of researchers created YOLOv4 [30] on the base of the darknet framework. Thereafter Glen Jocher introduced the latest version in the form of YOLOv5 using the PyTorch framework. YOLOv5 model is pre-trained on the COCO dataset, thus incorporating lessons and best practices evolved over the period of research and development.

YOLOv5 is made up of three parts Model backbone, Model neck, and Model head. Cross-Stage Partial Network (CSPNet) [31] is the backbone of the model. It helps reduce repeated gradient information and combines gradient change into a feature map. This results in the reduction of parameters and floating-point operation per second speed of inference of the model. Path Aggregation Network (PANet) [32] is applied at the neck to improve information flow. PANet is based on Feature Pyramid Network (FPN), which is a bottom-up path network [33]. A bottom-up path network improves the propagation of low-level features. Simultaneously, adaptive feature pooling, which connects the feature grid and all feature levels, is employed to ensure that important information in each feature level propagates straight to the next subnetwork. PANet optimises the exploitation of accurate localisation signals in lower layers, which can improve the object's position accuracy. To handle different sizes of objects, the head part of the YOLOv5 generates three different feature maps to achieve multi-scale prediction.

b. Faster R–CNN

**Fig. 7:**
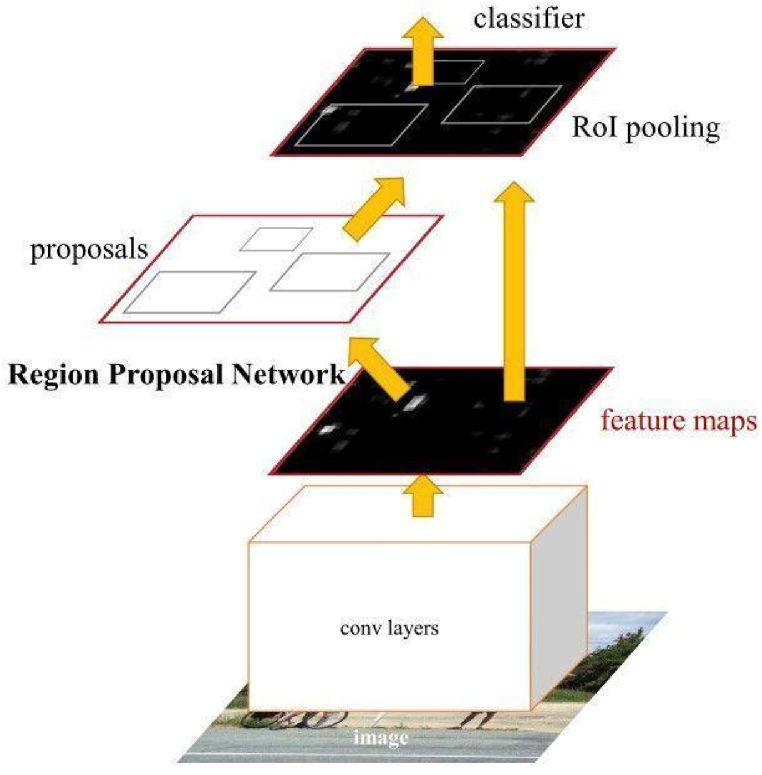
The architecture of Faster RCNN.

Faster RCNN is an extension to Fast RCNN with Region Proposal Network (RPN) added to it [5]. Fast RCNN uses selective search, whereas Faster RCNN uses RPN, which is faster. RPN ranks boxes in the region and proposes the most likely boxes that contain objects. Instead of using a pyramid of images, Faster RCNN uses anchor boxes for reference. The convolutional computation is shared between RPN and the FastRCNN. The architecture of Faster RCNN is shown in Fig. 7.

Firstly, the RPN generates region proposals, and for all region proposals in the image, using the Region of Interest (ROI) pooling layer, a feature vector is extracted. Then extracted feature vectors are then inferred, and bounding boxes are returned.

c. **Detectron2**

**Fig. 8:**
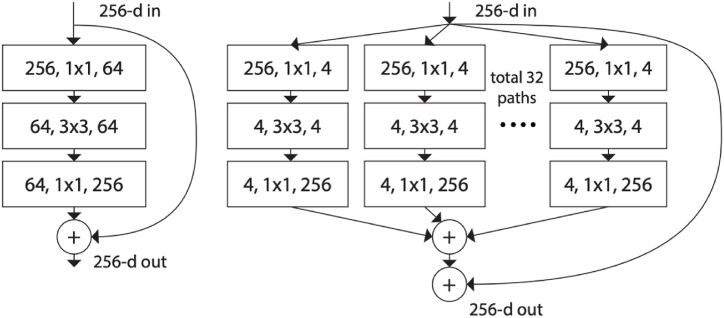
Difference between the ResNet and ResNeXt bottleneck blocks.

Detectron2 is a model of its own created by the Facebook AI Research FAIR group. It contains models that were present in the original Detectron. Detectron2 is written in PyTorch with an extra layer called Detectron2go [34]. In our experimentation, we used the ResNeXt-101-32 × 8d model from the framework [35]. The ResNeXt models are pre-trained on 940 million Instagram images with 1000 ImageNet1K synsets. After training, the models are fine-tuned on the ImageNet dataset [36]. It is based on the standard ResNet model, with 3 × 3 convolutions inside the bottleneck block replacing 3 × 3 grouped convolutions [37]. Fig. 8 shows the difference between the ResNet and ResNeXt bottleneck blocks. ResNeXt101-32 × 8d model's cardinality equals 32 and bottleneck width equals 8. The complete architecture of the ResNeXt-101-32 × 8d model with all of its layers can be referred here [38].

### 3.7 Dataset description

**Fig. 9:**
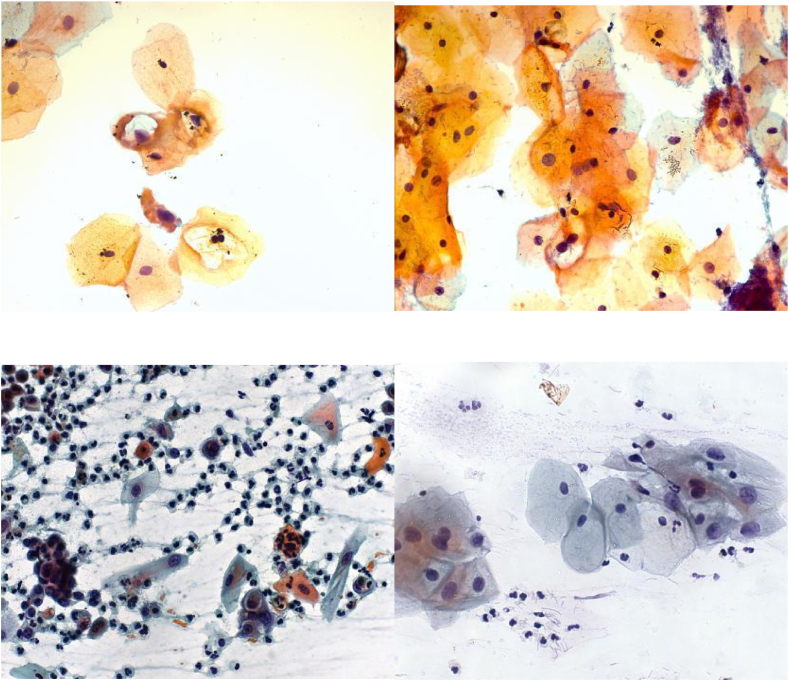
Sample images from the CRIC dataset.

The dataset is made ready for implementation in two steps- (1) Data acquisition from publicly available sources and (2) Data Pre-processing, which includes converting varying class datasets into two classes, namely Normal and Abnormal cells denoting non-cancerous and cancerous lesion presence, respectively. To have uniformity for comparison among the datasets, the multi-class dataset is converted into a binary class. The dataset used for the experimentation on the proposed model framework is the CRIC searchable image database created by the Center for Recognition and Inspection of Cells [21]. There are other datasets like Herlev and Sipakmed available, but they are not classified according to Bethesda System nomenclature, whereas the CRIC dataset follows Bethesda System, and the cells are classified following the rules by the cytopathologists. CRIC image database is one of the results of a diverse team of computer science and biology specialists. CRIC Cervix collection has in total of 400 images curated from conventional Pap smears, with the manual classification of 11,534 cells. Fig. 9 shows sample images from the CRIC dataset. These 11,534 cells are labelled in six classes, namely negative for intraepithelial lesion or malignancy (NILM); atypical squamous cells of undetermined significance, possibly non-neoplastic (ASC-US); low-grade squamous intraepithelial lesion (LSIL); atypical squamous cells, cannot exclude a high-grade lesion (ASC-H); high-grade squamous intraepithelial lesion (HSIL); and squamous cell carcinoma (SCC). For the two-class dataset, we combined ASC-US, SCC, LSIL, ASC-H, and HSIL into the Abnormal class, and NILM was converted into the Normal class. Table 3 shows the distribution of the dataset in 6 classes for train, valid, and test data with the number of cells in a class.

**Table 3:** Distribution of the dataset in 6 classes.

	ASC-H	ASC-US	HSIL	LSIL	NILM	SCC	Total
Train	667	501	1260	991	5443	136	8998
Valid	127	46	264	228	705	15	1385
Test	131	59	179	141	631	10	1151

## 4 Experimental setup

To prepare the dataset and to preprocess the images, we used the Roboflow tool [25]. Google colaboratory, a cloud-based service, is utilised for training and testing the suggested model framework [38]. Python 3 is utilised in Google colaboratory, along with supporting libraries like Tensorflow, Keras, and OpenCV. It includes a runtime GPU hardware accelerator for deep learning.

### 4.1 Results

Object detection models attempt to detect the existence of important things in pictures and classify those objects. The AP (Average Precision) measure is used to assess the accuracy of object detectors. The average precision (AP) is a method of condensing the accuracy-recall curve into a single number that represents the mean of all precisions. The difference between the current and next recalls is computed and then multiplied by the current precision using a loop that passes over all precisions/recalls. The AP is the weighted sum of precisions at each threshold, with the weight corresponding to the increase in recall. The formula for calculating AP is given in Equation (1). AP is the area under the precision-recall curve.

(1) $$AP=\sum_{k=0}^{k=n-1}\left[Recall\left(k\right)-Recall\left(k+1\right)\right]\times Precision\left(k\right)$$

where,

*n* = Number of thresholds, Recall(n) = 0, Precision(n)=1.

To train an object detection model, there are two inputs given, an image and ground-truth bounding boxes of the cells in the image. The object detection model predicts the bounding boxes of the detected object trying to match with the ground-truth boxes. A quantitative measure to score the match between ground truth and the predicted box is Intersection over Union (IoU). IoU is calculated using Equation (2).

(2) $$IoU=\frac{Intersection\;Area}{Union\;Area}$$

A threshold is set for IoU to the values 0.5 and 0.95 in classifying if the prediction is a true positive or false positive. IoU score defined the overlap; hence 0.5 score means that there is a 50 % overlap between predicted and ground-truth boxes. An AP is averaged over all categories in a multi-class dataset. This is called mean Average Precision (mAP). The mAP is calculated using Equation (3).

(3) $$mAP=\frac{1}{n}\sum_{k=1}^{k=n}{AP}_{k}$$

where

$${AP}_{k}=AP\;of\;class\;k$$

n=numberofclasses

mAP@[.5:.95] corresponds to the average AP for IoU from 0.5 to 0.95 with a step size of 0.05. Table 4 shows the AP score of three object detection models, namely YOLOv5, Faster RCNN, and Detectron2 with ResNeXt-101-32 × 8d for six classes on the dataset without augmentation. YOLOv5 generally performs better in terms of AP scores compared to Faster RCNN and Detectron2. Detectron2 has the highest AP scores for most classes, except for ASC-H, where YOLOv5 performs slightly better. For the class "LSIL," YOLOv5 has the highest AP score, indicating better performance in detecting this category. For the class "NILM," YOLOv5 shows significantly better performance with an AP score of 59.9 %, while Detectron2 with 51.30 %.The class "SCC" seems to be challenging for all models, as the AP scores are relatively low across the board.

**Table 4:** AP score of three object detection models, namely YOLOv5, Faster RCNN, and Detectron2 with ResNeXt-101-32 × 8d for six classes on the dataset without augmentation.

Model	ASC-H	ASC-US	HSIL	LSIL	NILM	SCC
YOLOv5	19.9	17.5	41.5	59.1	59.9	01.0
Faster RCNN	04.62	05.84	04.92	08.30	11.89	1.0
Detectron2	20.98	23.19	23.78	29.50	51.30	20.04

Table 5 shows the AP score of three object detection models, namely YOLOv5, Faster RCNN, and Detectron2 with ResNeXt-101-32 × 8d for six classes on the dataset with augmentation. YOLOv5 consistently outperforms Faster RCNN and Detectron2 across all classes, showing higher AP scores for each category. The class "NILM" (Negative for Intraepithelial Lesion or Malignancy) is relatively easy for all models to detect, as evident from the high AP scores for all three models. YOLOv5 has the highest AP score of 85.8 %, followed by Detectron2 with 46.23 % and Faster RCNN with 35.88 %. For the class "LSIL" (Low-Grade Squamous Intraepithelial Lesion), YOLOv5 has the lowest AP score among the classes, indicating that it might struggle more with detecting this category compared to the others. The class "SCC" (Squamous Cell Carcinoma) seems to be highly challenging for all models, as the AP scores are very low (around 1 %) for this category across all models.

**Table 5:** AP score of three object detection models, namely YOLOv5, Faster RCNN, and Detectron2 with ResNeXt-101-32 × 8d for six classes on the dataset with augmentation.

Model	ASC-H	ASC-US	HSIL	LSIL	NILM	SCC
YOLOv5	68.50	54.6	64.8	31.7	85.8	1.0
Faster RCNN	14.52	19.87	16.38	23.68	35.88	1.0
Detectron2	15.05	20.77	17.66	24.80	46.23	1.10

Table 6 shows the mAP score of the three models at the IoU threshold of 0.50 and in the range of 0.50–0.95 for all six classes, namely ASC-H, ASC-US, HSIL, LSIL, NILM, and SCC. YOLOv5 performs the best in terms of mAP without augmentation (40.60) and mAP with augmentation (54.80) on the dataset of 400 images. This suggests that YOLOv5 benefits from data augmentation techniques, as it shows a significant improvement in performance when augmenting the data.

**Table 6:** mAP score of the three models at the IoU threshold of 0.50 and in the range of 0.50–0.95 for all six classes, namely ASC-H, ASC-US, HSIL, LSIL, NILM, and SCC.

Models	mAP without augmentation on 400 images		mAP with augmentation on 400 images	
	mAP@.5	mAP@.5:.95	mAP@.5	mAP@.5:.95
YOLOv5	40.60	24.50	54.80	31.70
Faster R–CNN	12.50	04.80	33.80	17.70
Detectron2	46.40	30.8	36.00	24.00

Detectron2 has the highest mAP without augmentation (46.40), indicating better performance than both YOLOv5 and Faster R–CNN without data augmentation. However, its performance improvement with data augmentation (30.8) is not as significant as YOLOv5, as the mAP with augmentation for YOLOv5 is 54.80, which is considerably higher.

Faster R–CNN has the lowest mAP scores among the three models for both cases (with and without augmentation). This suggests that on this specific dataset, Faster R–CNN might not be as effective in detecting objects compared to YOLOv5 and Detectron2.

For mAP@.5, YOLOv5 (31.70) outperforms Faster R–CNN (17.70) and Detectron2 (24.00), indicating better accuracy in object detection when considering a lenient IoU threshold of 0.5.

For mAP@.5:.95, Detectron2 (24.00) performs slightly better than Faster R–CNN (17.70), while YOLOv5 (31.70) achieves the highest score, showing better performance across a range of IoU thresholds.

The models we implemented gave better results for two classes compared to the Bethesda nomenclature for six classes. The reason behind the better results in the two classes is due to the fact that the information-rich images are more when multiple classes of abnormal cells are combined to make one class named Abnormal.

Table 7 shows the AP score of three object detection models, namely YOLOv5, Faster RCNN, and Detectron2 with ResNeXt-101-32 × 8d for two classes on the dataset without augmentation. Both YOLOv5 and Detectron2 demonstrate high AP scores for the class "Normal," with 72 % and 50.15 %, respectively. This indicates that both models perform well in detecting instances of the "Normal" class in the dataset.

**Table 7:** AP score of three object detection models, namely YOLOv5, Faster RCNN, and Detectron2 with ResNeXt-101-32 × 8d for two classes on the dataset without augmentation.

Model	Normal	Abnormal
YOLOv5	72	71.7
Faster RCNN	48.61	25.62
Detectron2	50.15	42.19

For the class "Abnormal," YOLOv5 achieves a significantly higher AP score of 71.7 % compared to Faster R–CNN and Detectron2. This suggests that YOLOv5 excels in detecting instances of the "Abnormal" class, while the other two models struggle to achieve high precision and recall for this category.

Detectron2 performs better than Faster R–CNN in both classes, showing superior performance across the board. For example, Detectron2 achieves 42.19 % AP for the "Abnormal" class, while Faster R–CNN only achieves 25.62 %.

Overall, YOLOv5 demonstrates the best performance in this scenario, showing high AP scores for both classes. Detectron2 performs decently but falls behind YOLOv5, particularly in detecting the "Abnormal" class. Faster R–CNN lags in performance compared to both YOLOv5 and Detectron2.

Table 8 shows the AP score of three object detection models, namely YOLOv5, Faster RCNN, and Detectron2 with ResNeXt-101-32 × 8d for two classes on the dataset with augmentation. For the class "Normal," YOLOv5 achieves an AP score of 70.60 %, which is higher than both Faster R–CNN (46.8 %) and Detectron2 (43.94 %). This indicates that YOLOv5 is more effective in detecting instances of the "Normal" class in the dataset with augmentation.

**Table 8:** AP score of three object detection models, namely YOLOv5, Faster RCNN, and Detectron2 with ResNeXt-101-32 × 8d for two classes on the dataset with augmentation.

Model	Normal	Abnormal
YOLOv5	70.60	82.3
Faster RCNN	46.8	27.37
Detectron2	43.94	37.51

For the class "Abnormal," YOLOv5 performs exceptionally well with an AP score of 82.3 %. This score is significantly higher than both Faster R–CNN (27.37 %) and Detectron2 (37.51 %). YOLOv5 demonstrates superior performance in detecting instances of the "Abnormal" class compared to the other two models.

Comparing the three models for both classes, YOLOv5 consistently achieves the highest AP scores. This suggests that YOLOv5 with its architecture and the dataset augmentation is more capable of accurate and robust object detection, particularly for the given classes "Normal" and "Abnormal."

Detectron2 with ResNeXt-101-32 × 8d performs better than Faster R–CNN for both classes. For example, Detectron2 achieves an AP score of 37.51 % for the "Abnormal" class, while Faster R–CNN only achieves 27.37 %.

Table 9 shows the mAP score of the three models at the IoU threshold of 0.50 and in the range of 0.50–0.95 for all two classes, namely Normal and Abnormal, on the dataset with augmentation. YOLOv5 achieves the highest mAP score without augmentation (82.6 %), suggesting that it performs very well on the dataset of 400 images without the use of data augmentation techniques. Detectron2 follows with an mAP of 76 %, which is also quite high, indicating good performance even without data augmentation. Faster R–CNN lags behind with an mAP of 55.4 %, suggesting that it may not perform as well as the other two models on the original dataset.

**Table 9:** mAP score of the three models at the IoU threshold of 0.50 and in the range of 0.50–0.95 for all two classes, namely Normal and Abnormal, on the dataset with augmentation.

Models	mAP without augmentation on 400 images		mAP with augmentation on 400 images	
	mAP@.5	mAP@.5:.95	mAP@.5	mAP@.5:.95
YOLOv5	82.6	52.3	83	53.7
Faster R–CNN	55.4	28.80	52.8	26.0.
Detectron2	76	47.6	72.60	44.23

For this metric, YOLOv5 obtains the highest mAP of 52.3 %, although it's lower than its performance without augmentation. This suggests that data augmentation might not provide a significant improvement for YOLOv5 on this dataset. Detectron2 follows with an mAP of 47.6 %, which is also lower than its performance without augmentation. Faster R–CNN again has the lowest mAP (28.80 %), indicating that data augmentation might not have a significant impact on its performance as well.

YOLOv5 achieves the highest mAP when considering an IoU threshold of 0.50 (83 %), showing its strength in detecting objects with a relatively lenient overlap. Detectron2 follows with an mAP of 72.60 % at the same IoU threshold, indicating good performance in detecting objects with a 0.50 IoU overlap. Faster R–CNN obtains an mAP of 52.8 % at the IoU threshold of 0.50, which is lower compared to the other two models.

YOLOv5 continues to show strong performance with an mAP of 53.7 % when considering a range of IoU thresholds from 0.50 to 0.95. Detectron2 follows with an mAP of 44.23 %, indicating that it still performs reasonably well across a range of IoU thresholds. Faster R–CNN has the lowest mAP at 26.0 %, suggesting that it struggles to achieve high precision and recall across the range of IoU thresholds.

**Fig. 10:**
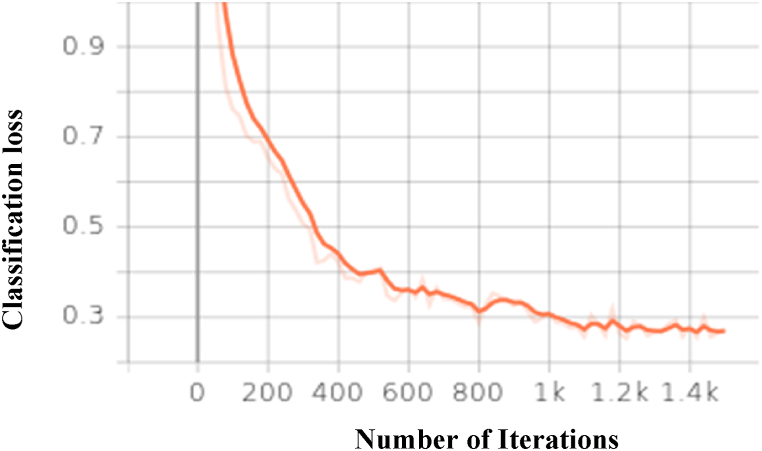
Classification loss for Detectron2 ResNeXt-101-32 × 8d over the 1500 iterations

The graph in Fig. 10 shows classification loss for Detectron2 ResNeXt-101-32 × 8d over the 1500 iterations. The figure shows the loss for Detectron2 ResNeXt-101-32 × 8d with respect to number of epochs. It seen that as the training progress with increasing number of epochs loss getting reduced.

**Fig. 11:**
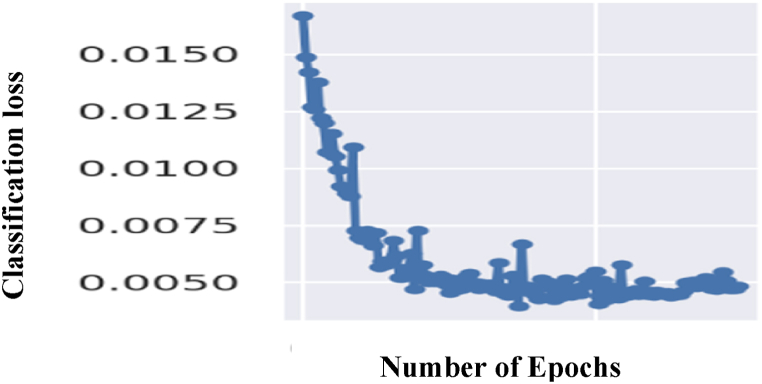
Classification loss for YOLOv5 over the 150 epochs

The graph in Fig. 11 shows the classification loss for YOLOv5 over the 150 epochs. It can be observed that as the number of epochs increases, the loss is reduced, indicating better training of the model.

**Fig. 12:**
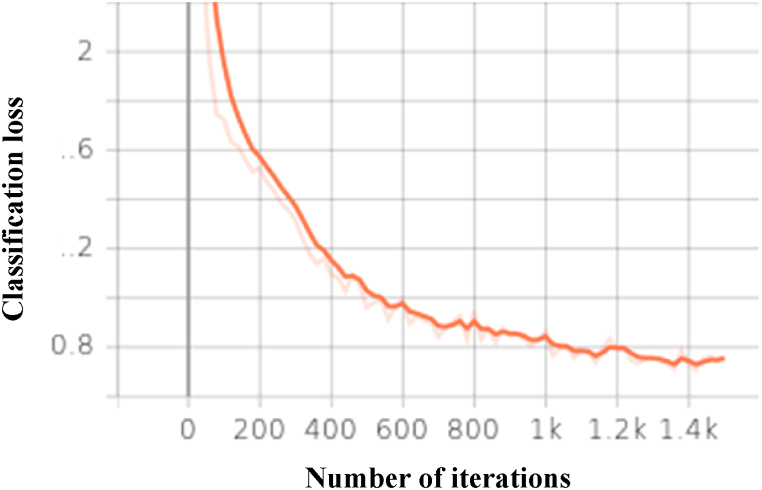
Classification loss for Faster RCNN over the 1500 steps.

The graph in Fig. 12 shows the classification loss for Faster RCNN over the 1500 steps. The value of the loss is dropped as the number of epochs is increased for the Faster RCNN.

**Fig. 13:**
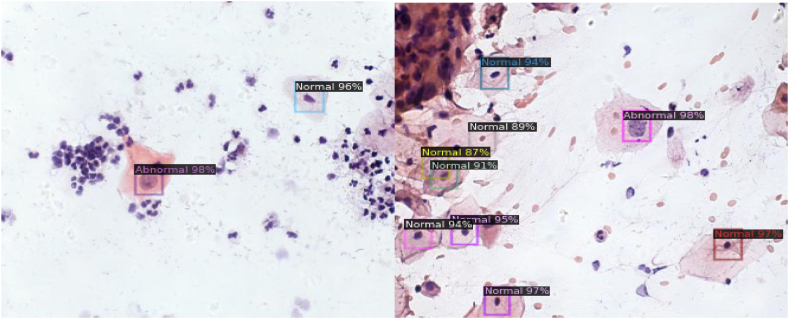
Prediction of Detectron2 ResnExt-101-32 × 8d on binary classes.

Fig. 13 below shows the prediction of Detectron2 ResnExt-101-32 × 8d on binary classes, namely Normal and Abnormal. The result image displays the confidence score along with the prediction. The model predicts a bounding box around the nucleus of the cell. From the results, we can see that our model can differentiate between cells and other impurities present in the sample.

**Fig. 14:**
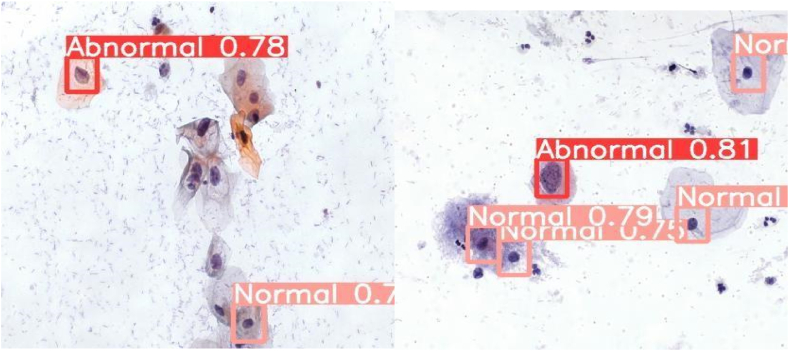
prediction of YOLOv5 on binary classes.

Fig. 14 below shows the prediction of YOLOv5 on binary classes, namely Normal and Abnormal.

**Fig. 15:**
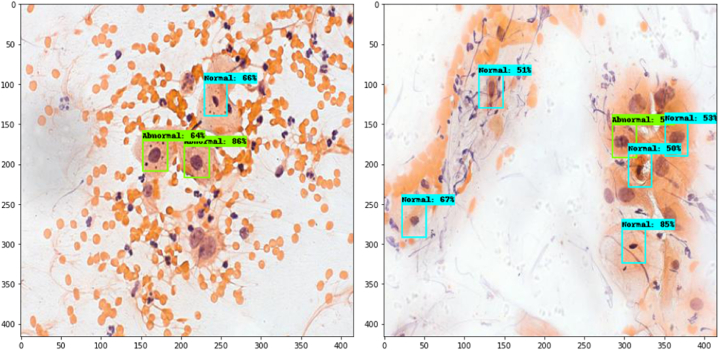
Prediction of FasterRCNN on binary classes.

Fig. 15 below shows the prediction of Faster RCNN on binary classes, namely Normal and Abnormal. It can be clearly observed that the model has correctly detected the normal and abnormal cells in the image.

## 5 Conclusion and future work

The experiments presented in this paper show that object detection can help in detecting and identifying cancerous cells into classes, as noted in the Bethesda nomenclature system. In this paper, we talked about the latest YOLOv5 model, Faster RCNN, and Detectron framework with the ResNeXt101-32 × 8d model. With state-of-the-art models and frameworks, we achieved the highest detection mAPof 82.4 % at a threshold of 50 % on IoU using the YOLOv5 model on 2 class object detector. Object Detection prediction is better when we have two classes compared to 6 classes. Compared to object detection models discussed in the literature survey, the implemented models gave better cervical cancer cell prediction, but more work can be done to increase the accuracy of the models and achieve a higher mAP than 83 %. Multi-class cervical cancer cell prediction is an area where with more number of different samples, we can create a model that can match and exceed the prediction of binary classification between Normal and Abnormal.

CRIC dataset used for training the models may not fully represent the diversity and variability of real-world pap smear images. A small dataset may lead to limited generalization and may not cover all possible variations in cervical cells. The CRIC dataset has conventional pap smear samples; since our models are generic in nature, similar models can be implemented for Liquid-Based-Cytology (LBC) pap smear samples. Along with this, pap smear images contain a lot of distortion in image quality. Improving image quality without losing morphological information can be implemented to improve the accuracy of the models. State-of-the-art object detection models like YOLOv5 and Faster R–CNN and Detectron are computationally intensive and require powerful hardware for training and inference.

## Data availability

Data will be made available on request.

## CRediT authorship contribution statement

**Madhura Kalnhor:** Writing – original draft, Methodology, Investigation. **Swati Shinde:** Writing – review & editing, Supervision, Funding acquisition, Conceptualization. **Pankaj Wajire:** Writing – original draft, Validation, Methodology, Investigation. **Hemanth Jude:** Supervision, Conceptualization, Writing - review & editing.

## Declaration of competing interest

The authors declare the following financial interests/personal relationships which may be considered as potential competing interests:Dr. Swati Shinde reports was provided by Pimpri Chinchwad College of Engineering. If there are other authors, they declare that they have no known competing financial interests or personal relationships that could have appeared to influence the work reported in this paper.
